# 2,6-Difluoro­benzoic acid

**DOI:** 10.1107/S1600536810028758

**Published:** 2010-07-24

**Authors:** Mohammad T. M. Al-Dajani, Habibah A Wahab, Nornisah Mohamed, Chin Sing Yeap, Hoong-Kun Fun

**Affiliations:** a School of Pharmaceutical Sciences, Universiti Sains Malaysia, 11800 USM, Penang, Malaysia; b Malaysian Institute of Pharmaceuticals and Nutraceuticals, Ministry of Science, Technology and Innovation, Blok A, 10 Persiaran Bukit Jambul, 11900 Bayan Lepas, Penang, Malaysia; c X-ray Crystallography Unit, School of Physics, Universiti Sains Malaysia, 11800 USM, Penang, Malaysia

## Abstract

In the title compound, C_7_H_4_F_2_O_2_, the dihedral angle between the benzene ring and the carboxyl­ate group is 33.70 (14)°. In the crystal structure, inversion dimers linked by pairs of O—H⋯O hydro­gren bonds occur, generating *R*_2_^2^(8) loops. The dimers are linked into sheets lying parallel to (102) by C—H⋯F hydrogen bonds.

## Related literature

For general background to 2,6-diflorobenzyl­chloride derivatives, see: Beavo (1995 ▶); Beavo & Reifsnyder (1990 ▶); Nicholson *et al.* (1991 ▶). For the stability of the temperature controller used in the data collection, see: Cosier & Glazer (1986 ▶).
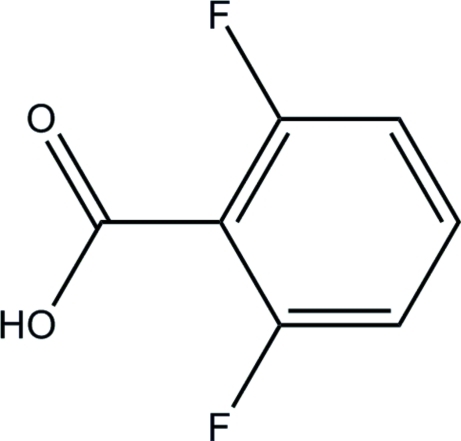


## Experimental

### 

#### Crystal data

C_7_H_4_F_2_O_2_
*M* *_r_* = 158.10
Monoclinic, 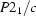

*a* = 3.6517 (4) Å
*b* = 14.1214 (15) Å
*c* = 12.2850 (13) Å
β = 95.651 (3)°
*V* = 630.42 (12) Å^3^
*Z* = 4
Mo *K*α radiation
μ = 0.16 mm^−1^
*T* = 100 K
0.73 × 0.19 × 0.09 mm

#### Data collection

Bruker APEXII DUO CCD diffractometer
Absorption correction: multi-scan (*SADABS*; Bruker, 2009 ▶) *T*_min_ = 0.841, *T*_max_ = 0.986
6112 measured reflections
2190 independent reflections
1895 reflections with *I* > 2σ(*I*)
*R*_int_ = 0.029

#### Refinement

*R*[*F* ^2^ > 2σ(*F* ^2^)] = 0.049
*wR*(*F* ^2^) = 0.143
*S* = 1.12
2190 reflections
116 parameters
All H-atom parameters refined
Δρ_max_ = 0.47 e Å^−3^
Δρ_min_ = −0.31 e Å^−3^

### 

Data collection: *APEX2* (Bruker, 2009 ▶); cell refinement: *SAINT* (Bruker, 2009 ▶); data reduction: *SAINT*; program(s) used to solve structure: *SHELXTL* (Sheldrick, 2008 ▶); program(s) used to refine structure: *SHELXTL*; molecular graphics: *SHELXTL*; software used to prepare material for publication: *SHELXTL* and *PLATON* (Spek, 2009 ▶).

## Supplementary Material

Crystal structure: contains datablocks global, I. DOI: 10.1107/S1600536810028758/hb5558sup1.cif

Structure factors: contains datablocks I. DOI: 10.1107/S1600536810028758/hb5558Isup2.hkl

Additional supplementary materials:  crystallographic information; 3D view; checkCIF report

## Figures and Tables

**Table 1:** Hydrogen-bond geometry (Å, °)

*D*—H⋯*A*	*D*—H	H⋯*A*	*D*⋯*A*	*D*—H⋯*A*
O2—H1*O*2⋯O3^i^	0.95 (4)	1.68 (4)	2.6318 (14)	174 (4)
C3—H3⋯F2^ii^	0.98 (2)	2.54 (2)	3.3428 (16)	138.7 (16)

Symmetry codes: (i) 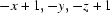; (ii) 
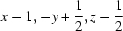
.

**Fig. 1.**
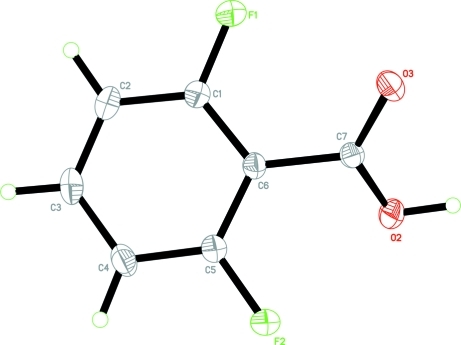
The molecular structure of (I) with 50% probability ellipsoids for non-H atoms.

**Fig. 2.**
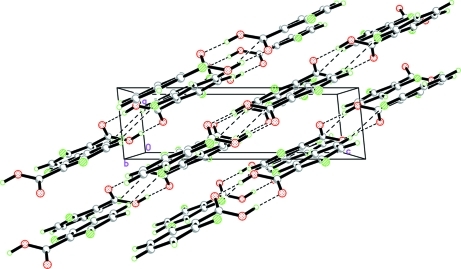
The crystal packing of (I), viewed down the b axis, showing two 2-D planes.

## supplementary crystallographic information

### Comment

The derivatives of 2,6-diflorobenzylchloride involved in the inhibition of phosphodiesterases (PDEs) are enzymes which catalyze PDEs. These derivatives are classified into seven families, five of which, PDE1–PDE5, have been characterized (Beavo, 1995). The hydrolysis of cyclic nucleotides was evaluated according to the methods in given the references (Beavo & Reifsnyder, 1990; Nicholson *et al.*, 1991).

The molecule of the title compound, (I), (Fig. 1) is not planar with the dihedral angle between the benzene ring and the carboxylate group being 33.70 (14)°. In the crystal structure, the molecules are linked into pairs of centrosymmetric dimers by intermolecular O2—H1O2···O3 hydrogren bonds (Table 1). These dimers are linked into two-dimensional plane by the intermolecular C3—H3A···F2 hydrogen bonds (Fig. 2, Table 1) parallel to (102).

### Experimental

2,6-Difluorobenzylchloride (0.01 mol, 1.7 g) was added drop-wise with stirring into a round bottom flask containing 25 ml water and then refluxed for two and half hours. The gum compound precipitate formed was filtered and dissolved in alkaline water. Hydrochloric acid was then added drop-wise with stirring. The white precipitate formed was dissolved in methanol. Colourless needles of (I) were formed at room temperature overnight and filtrated and dried at 333 K.

### Refinement

All hydrogen atoms were located in a difference Fourier map and refined freely.

### Crystal data

**Table:**

C_7_H_4_F_2_O_2_	*F*(000) = 320
*M**_r_* = 158.10	*D*_x_ = 1.666 Mg m^−^^3^
Monoclinic, *P*2_1_/*c*	Mo *K*α radiation, λ = 0.71073 Å
Hall symbol: -P 2ybc	Cell parameters from 3166 reflections
*a* = 3.6517 (4) Å	θ = 3.3–32.1°
*b* = 14.1214 (15) Å	µ = 0.16 mm^−^^1^
*c* = 12.2850 (13) Å	*T* = 100 K
β = 95.651 (3)°	Needle, colourless
*V* = 630.42 (12) Å^3^	0.73 × 0.19 × 0.09 mm
*Z* = 4	

### Data collection

**Table:**

Bruker APEXII DUO CCD diffractometer	2190 independent reflections
Radiation source: fine-focus sealed tube	1895 reflections with *I* > 2σ(*I*)
graphite	*R*_int_ = 0.029
φ and ω scans	θ_max_ = 32.1°, θ_min_ = 2.2°
Absorption correction: multi-scan (*SADABS*; Bruker, 2009)	*h* = −5→5
*T*_min_ = 0.841, *T*_max_ = 0.986	*k* = −20→20
6112 measured reflections	*l* = −18→18

### Refinement

**Table:**

Refinement on *F*^2^	Primary atom site location: structure-invariant direct methods
Least-squares matrix: full	Secondary atom site location: difference Fourier map
*R*[*F*^2^ > 2σ(*F*^2^)] = 0.049	Hydrogen site location: inferred from neighbouring sites
*wR*(*F*^2^) = 0.143	All H-atom parameters refined
*S* = 1.12	*w* = 1/[σ^2^(*F*_o_^2^) + (0.0668*P*)^2^ + 0.3079*P*] where *P* = (*F*_o_^2^ + 2*F*_c_^2^)/3
2190 reflections	(Δ/σ)_max_ < 0.001
116 parameters	Δρ_max_ = 0.47 e Å^−^^3^
0 restraints	Δρ_min_ = −0.31 e Å^−^^3^

### Special details

**Table:**

Experimental. The crystal was placed in the cold stream of an Oxford Cryosystems Cobra open-flow nitrogen cryostat (Cosier & Glazer, 1986) operating at 100.0 (1) K.
Geometry. All e.s.d.'s (except the e.s.d. in the dihedral angle between two l.s. planes) are estimated using the full covariance matrix. The cell e.s.d.'s are taken into account individually in the estimation of e.s.d.'s in distances, angles and torsion angles; correlations between e.s.d.'s in cell parameters are only used when they are defined by crystal symmetry. An approximate (isotropic) treatment of cell e.s.d.'s is used for estimating e.s.d.'s involving l.s. planes.
Refinement. Refinement of *F*^2^ against ALL reflections. The weighted *R*-factor *wR* and goodness of fit *S* are based on *F*^2^, conventional *R*-factors *R* are based on *F*, with *F* set to zero for negative *F*^2^. The threshold expression of *F*^2^ > σ(*F*^2^) is used only for calculating *R*-factors(gt) *etc*. and is not relevant to the choice of reflections for refinement. *R*-factors based on *F*^2^ are statistically about twice as large as those based on *F*, and *R*- factors based on ALL data will be even larger.

### Fractional atomic coordinates and isotropic or equivalent isotropic displacement parameters (Å^2^)

**Table:**

	*x*	*y*	*z*	*U*_iso_*/*U*_eq_	
F1	0.0410 (3)	−0.01635 (6)	0.16839 (7)	0.0312 (2)	
F2	0.2348 (3)	0.26707 (6)	0.36896 (7)	0.0285 (2)	
O2	0.2287 (3)	0.09843 (7)	0.46658 (7)	0.0238 (2)	
O3	0.4751 (3)	−0.00746 (7)	0.35958 (8)	0.0222 (2)	
C1	0.0163 (4)	0.07830 (9)	0.17659 (9)	0.0194 (2)	
C2	−0.1413 (4)	0.12763 (10)	0.08679 (10)	0.0228 (3)	
C3	−0.1734 (4)	0.22519 (10)	0.09441 (10)	0.0234 (3)	
C4	−0.0482 (4)	0.27250 (10)	0.19005 (11)	0.0230 (3)	
C5	0.1044 (4)	0.22003 (9)	0.27793 (9)	0.0184 (2)	
C6	0.1398 (3)	0.12160 (8)	0.27576 (9)	0.0160 (2)	
C7	0.2939 (3)	0.06665 (8)	0.37288 (9)	0.0156 (2)	
H2	−0.221 (7)	0.0927 (16)	0.0182 (18)	0.038 (6)*	
H3	−0.285 (6)	0.2599 (15)	0.0302 (17)	0.035 (5)*	
H4	−0.074 (6)	0.3406 (16)	0.1979 (18)	0.035 (5)*	
H1O2	0.341 (11)	0.062 (3)	0.526 (3)	0.098 (12)*	

### Atomic displacement parameters (Å^2^)

**Table:**

	*U*^11^	*U*^22^	*U*^33^	*U*^12^	*U*^13^	*U*^23^
F1	0.0518 (6)	0.0183 (4)	0.0219 (4)	0.0019 (4)	−0.0044 (4)	−0.0039 (3)
F2	0.0459 (6)	0.0169 (4)	0.0210 (4)	−0.0010 (4)	−0.0048 (4)	−0.0018 (3)
O2	0.0340 (6)	0.0238 (5)	0.0135 (4)	0.0038 (4)	0.0014 (4)	0.0013 (3)
O3	0.0269 (5)	0.0179 (4)	0.0216 (4)	0.0056 (4)	0.0012 (4)	0.0030 (3)
C1	0.0238 (6)	0.0181 (5)	0.0162 (5)	0.0001 (4)	0.0014 (4)	0.0003 (4)
C2	0.0242 (6)	0.0289 (6)	0.0150 (5)	−0.0002 (5)	−0.0006 (4)	0.0019 (4)
C3	0.0224 (6)	0.0289 (6)	0.0184 (5)	0.0037 (5)	−0.0001 (4)	0.0078 (4)
C4	0.0272 (6)	0.0193 (6)	0.0225 (6)	0.0041 (5)	0.0016 (5)	0.0062 (4)
C5	0.0210 (5)	0.0174 (5)	0.0166 (5)	0.0004 (4)	0.0012 (4)	0.0011 (4)
C6	0.0182 (5)	0.0160 (5)	0.0137 (4)	0.0009 (4)	0.0014 (4)	0.0019 (3)
C7	0.0174 (5)	0.0148 (5)	0.0148 (4)	−0.0006 (4)	0.0020 (4)	0.0012 (3)

### Geometric parameters (Å, °)

**Table:**

F1—C1	1.3442 (15)	C2—H2	0.99 (2)
F2—C5	1.3467 (14)	C3—C4	1.3892 (19)
O2—C7	1.2794 (14)	C3—H3	0.98 (2)
O2—H1O2	0.96 (4)	C4—C5	1.3807 (17)
O3—C7	1.2574 (15)	C4—H4	0.97 (2)
C1—C2	1.3815 (17)	C5—C6	1.3965 (17)
C1—C6	1.3976 (16)	C6—C7	1.4866 (15)
C2—C3	1.387 (2)		
			
C7—O2—H1O2	113 (2)	C5—C4—H4	119.3 (13)
F1—C1—C2	117.86 (11)	C3—C4—H4	122.2 (13)
F1—C1—C6	118.83 (11)	F2—C5—C4	117.84 (11)
C2—C1—C6	123.29 (12)	F2—C5—C6	118.74 (10)
C1—C2—C3	118.58 (12)	C4—C5—C6	123.38 (11)
C1—C2—H2	119.4 (13)	C5—C6—C1	115.44 (10)
C3—C2—H2	122.0 (13)	C5—C6—C7	122.18 (10)
C2—C3—C4	120.79 (11)	C1—C6—C7	122.37 (11)
C2—C3—H3	118.2 (13)	O3—C7—O2	123.76 (11)
C4—C3—H3	121.0 (13)	O3—C7—C6	119.51 (10)
C5—C4—C3	118.49 (12)	O2—C7—C6	116.72 (10)
			
F1—C1—C2—C3	179.13 (13)	C4—C5—C6—C7	−177.92 (12)
C6—C1—C2—C3	1.2 (2)	F1—C1—C6—C5	−179.87 (12)
C1—C2—C3—C4	0.3 (2)	C2—C1—C6—C5	−1.92 (19)
C2—C3—C4—C5	−0.9 (2)	F1—C1—C6—C7	−0.65 (19)
C3—C4—C5—F2	178.01 (12)	C2—C1—C6—C7	177.31 (12)
C3—C4—C5—C6	0.0 (2)	C5—C6—C7—O3	−147.25 (13)
F2—C5—C6—C1	−176.65 (11)	C1—C6—C7—O3	33.57 (18)
C4—C5—C6—C1	1.31 (19)	C5—C6—C7—O2	33.33 (17)
F2—C5—C6—C7	4.12 (18)	C1—C6—C7—O2	−145.84 (13)

### Hydrogen-bond geometry (Å, °)

**Table:**

*D*—H···*A*	*D*—H	H···*A*	*D*···*A*	*D*—H···*A*
O2—H1O2···O3^i^	0.95 (4)	1.68 (4)	2.6318 (14)	174 (4)
C3—H3···F2^ii^	0.98 (2)	2.54 (2)	3.3428 (16)	138.7 (16)

Symmetry codes: (i) −*x*+1, −*y*, −*z*+1; (ii) *x*−1, −*y*+1/2, *z*−1/2.
